# Circulating microRNAs predispose to takotsubo syndrome following high-dose adrenaline exposure

**DOI:** 10.1093/cvr/cvab210

**Published:** 2021-06-22

**Authors:** Liam S Couch, Jan Fiedler, Giles Chick, Rory Clayton, Eef Dries, Laura M Wienecke, Lu Fu, Jerome Fourre, Pragati Pandey, Anselm A Derda, Brian X Wang, Richard Jabbour, Mayooran Shanmuganathan, Peter Wright, Alexander R Lyon, Cesare M Terracciano, Thomas Thum, Sian E Harding

**Affiliations:** National Heart and Lung Institute, Imperial College London, London, UK; Institute of Molecular and Translational Therapeutic Strategies, Hannover Medical School, Hannover, Germany; Institute of Molecular and Translational Therapeutic Strategies, Hannover Medical School, Hannover, Germany; Fraunhofer Institute for Toxicology and Experimental Medicine (ITEM), Hannover, Germany; National Heart and Lung Institute, Imperial College London, London, UK; National Heart and Lung Institute, Imperial College London, London, UK; National Heart and Lung Institute, Imperial College London, London, UK; National Heart and Lung Institute, Imperial College London, London, UK; Institute of Molecular and Translational Therapeutic Strategies, Hannover Medical School, Hannover, Germany; Department of Cardiology and Angiology, Hannover Medical School, Hannover, Germany; National Heart and Lung Institute, Imperial College London, London, UK; Department of Physiology, Xuzhou Medical University, Xuzhou, Jiangsu Province, China; National Heart and Lung Institute, Imperial College London, London, UK; National Heart and Lung Institute, Imperial College London, London, UK; National Heart and Lung Institute, Imperial College London, London, UK; Institute of Molecular and Translational Therapeutic Strategies, Hannover Medical School, Hannover, Germany; Department of Cardiology and Angiology, Hannover Medical School, Hannover, Germany; National Heart and Lung Institute, Imperial College London, London, UK; National Heart and Lung Institute, Imperial College London, London, UK; National Heart and Lung Institute, Imperial College London, London, UK; National Heart and Lung Institute, Imperial College London, London, UK; Department of Life Sciences, University of Roehampton, London, UK; National Heart and Lung Institute, Imperial College London, London, UK; Department of Cardiology, Royal Brompton Hospital, London, UK; National Heart and Lung Institute, Imperial College London, London, UK; National Heart and Lung Institute, Imperial College London, London, UK; Institute of Molecular and Translational Therapeutic Strategies, Hannover Medical School, Hannover, Germany; Fraunhofer Institute for Toxicology and Experimental Medicine (ITEM), Hannover, Germany; National Heart and Lung Institute, Imperial College London, London, UK

**Keywords:** Takotsubo syndrome, Adrenaline, MicroRNAs, *In vivo*, Cardiomyocyte, Heart failure, Stress

## Abstract

**Aims:**

Takotsubo syndrome (TTS) is an acute heart failure, typically triggered by high adrenaline during physical or emotional stress. It is distinguished from myocardial infarction (MI) by a characteristic pattern of ventricular basal hypercontractility with hypokinesis of apical segments, and in the absence of culprit coronary occlusion. We aimed to understand whether recently discovered circulating biomarkers miR-16 and miR-26a, which differentiate TTS from MI at presentation, were mechanistically involved in the pathophysiology of TTS.

**Methods and results:**

miR-16 and miR-26a were co-overexpressed in rats with AAV and TTS induced with an adrenaline bolus. Untreated isolated rat cardiomyocytes were transfected with pre-/anti-miRs and functionally assessed. Ventricular basal hypercontraction and apical depression were accentuated in miR-transfected animals after induction of TTS. *In vitro* miR-16 and/or miR-26a overexpression in isolated apical (but not basal), cardiomyocytes produced strong depression of contraction, with loss of adrenaline sensitivity. They also enhanced the initial positive inotropic effect of adrenaline in basal cells. Decreased contractility after TTS-miRs was reproduced in non-failing human apical cardiomyocytes. Bioinformatic profiling of miR targets, followed by expression assays and functional experiments, identified reductions of CACNB1 (L-type calcium channel Ca_v_β subunit), RGS4 (regulator of G-protein signalling 4), and G-protein subunit Gβ (GNB1) as underlying these effects.

**Conclusion:**

miR-16 and miR-26a sensitize the heart to TTS-like changes produced by adrenaline. Since these miRs have been associated with anxiety and depression, they could provide a mechanism whereby priming of the heart by previous stress causes an increased likelihood of TTS in the future.

## Graphical Abstract

**Graphical Abstract:**
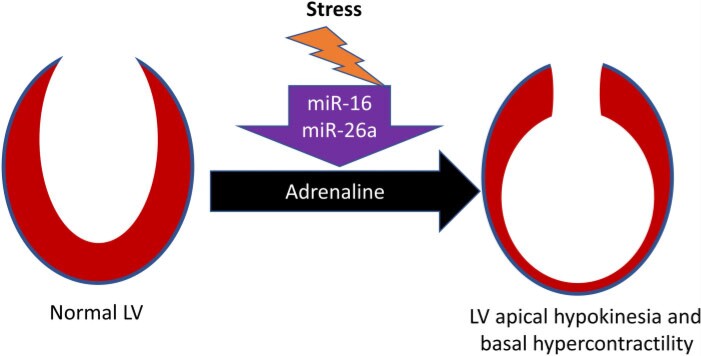

## Introduction

Takotsubo syndrome (TTS), colloquially known as *broken heart syndrome*, is a severe but reversible acute heart failure^1^ that occurs following a catecholamine surge.^2^ TTS predominantly affects post-menopausal women, and typically results from extreme physical or emotional stress.^3,4^ TTS patients acutely resemble those with myocardial infarction (MI), presenting with chest pain and ST-segment elevation on electrocardiogram, but are distinguished by a characteristic pattern of left ventricular apical hypokinesia with basal hypercontractility, occurring in the absence of culprit coronary artery disease.^3^ TTS is estimated to represent 5–6% of female patients presenting with suspected STEMI.^5^ The profound contractile dysfunction in TTS causes serious complications, including cardiogenic shock, thrombi, LV rupture, pulmonary oedema, and arrhythmia. This results in an acute mortality rate of 4–5%,^6^ similar to MI.^5^ Although recovery for surviving patients is usually within weeks, long-term contractile dysfunction can occur, particularly with exercise,^7^ and long-term outcomes are comparable to patients with acute coronary syndrome.^8^ There is no evidence-based treatment for the acute or chronic management of TTS,^9^ therefore a greater understanding TTS pathogenesis is important.

The association of TTS with catecholamines is well-evidenced, although several pathophysiological hypotheses exist, including direct catecholaminergic myocardial stunning and microvascular endothelial dysfunction. Furthermore, the brain–heart interaction within TTS is now becoming appreciated with changes in the autonomic nervous system (ANS)and limbic system.^10,11^

Preclinical rodent models induce TTS *in vivo* by bolus injection of adrenaline or isoprenaline,^12,13^ and have been frequently used to investigate the pathophysiology of TTS. These stimulate both β_1_AR and β_2_ARs, and while β_1_AR only signals via the canonical stimulatory G-protein (Gαs), β_2_AR can signal via Gαs or the inhibitory G-protein (Gαi). Physiologically, this limits toxicity of high catecholamines by shifting receptor coupling from Gαs to Gαi, known as stimulus trafficking. This reverses Gαs positive inotropy and is directly cardiodepressive. Depression of contraction is amplified at the apex due to the increased proportion of β_2_ARs, producing the characteristic apical ballooning.^12,14^ While Gαi inhibition in rodents reverses the contractile changes of TTS, it also increases mortality from arrhythmic sudden death, hence the hypothesis that Gαi is linked to a cardioprotective pathway.^12,13^

Recently, circulating microRNA (miR)-16 and miR-26a have been identified as specific biomarkers which are increased in TTS *versus* STEMI or healthy controls.^15^ miR-1 and miR-133a were found to be largely increased in STEMI vs. TTS, although were raised in both vs. control. Since miR-1 and miR-133a are cardiac-enriched,^16^ they likely reflect the degree of myocardial damage that has occurred, being more modestly raised in TTS than in STEMI, as occurs with cardiac troponin and creatine kinase-MB. Since miR-16 and miR-26a are specifically increased in TTS, they were selected for further investigation as it is not known whether they have a causal relation to TTS or simply represent catecholamine activation/damage. The organ or tissue of origin of miR-16 and miR-26a in TTS is similarly unknown, since they were originally identified in circulating blood of TTS patients. miR-16 and miR-26a have been linked to stress, depression and anxiety^17–19^ and TTS has a strong correlation with pre-existing psychiatric disorders including anxiety and depression.^3^ These miRs could therefore represent novel effectors in the brain–heart interaction within TTS. Here, we have shown that miR-16 and miR-26a accentuate the TTS-like changes in contractility observed in TTS in rat (*in vivo* and *in vitro*) and in human cardiomyocytes, and identified the mechanisms underlying these effects.

## Methods

All procedures followed the standards for the care and use of animal subjects as stated in the Guide of the Care and Use of Laboratory Animals (NIH publication No. 85-23, revised 1996) and the requirements of the UK Home Office (ASPA 1986 Amendments Regulations 2012) incorporating the EU directive 2010/63/EU. Protocols were approved by the Animal Welfare and Ethical Review Board of Imperial College London.

### 2.1 In vivo takotsubo syndrome model in AAV9-treated rats

Adult male Sprague–Dawley rats (75–150 g) were infected with AAV9-EF1α-mCherry (AAV-control) or AAV9-EF1α-pri-miR-16+pri-miR-26a-mCherry (AAV-miR) at 2.5 × 10^12^ gc via tail vein injection. After 6 weeks, TTS was induced as previously described,^12^ by bolus injection of adrenaline through the external jugular vein. Animals were anaesthetised with continuous inhaled isoflurane for initial induction of anaesthesia and subsequent maintenance for the procedure (5% isoflurane in 5 L/min 100% O_2_ and 1.5–2.5% isoflurane in 1.5 L/min 100% O_2_, respectively).

The adrenaline method for induction of TTS was chosen as opposed to the isoprenaline method in this case, because the compressed timescale concentrates on the immediate contractile changes. In this cohort, adrenaline concentration was reduced by a half-log unit in molarity compared to the initial study (from 55 to 18 µg/kg). This was a practical decision since the AAV-treated rats were heavier, with increased body fat percentage, after 6 weeks, and were more sensitive to arrhythmic sudden death. However, it was a serendipitous choice, since it clearly showed the TTS-enhancing effect of miR pre-treatment. Regional LV contractility was quantified from M-mode echocardiographic recordings (Vevo 770, Visualsonics) using a fixed parasternal long-axis view. Percentage fractional shortening (FS) was measured at the apex, mid-LV, and base at baseline, and every 5 min following adrenaline infusion. These data were double-blinded and analysed by an independent expert. All rats were included. Rats were euthanisedunder isoflurane anaesthesia (as previously described) by a non-schedule one method of excising the heart according to the approved protocol. Excised ventricles were divided into five transverse segments from apex to base using a controlled device, discarding the middle section and utilizing the upper two sections for base and lower two for apex for fixation and RNA/protein quantification.

### 2.2 Monitoring of AAV-treated rats

After consulting a veterinarian from Imperial CBS, a behavioural monitoring plan was devised. The Rat Grimace Scale^20^ was used daily to monitor whether the AAV treated rats experienced any pain during the 6-week monitoring period. The activity level of the rats was evaluated with the help of an experienced animal technician, blinded to treatment group, to grade the average activity level of a cage of animals treated with either AAV-control or AAV-miR. Low, medium, and high levels of activity were assigned values of 0, 1, and 2. AAV-treated rats were weighed at baseline and then weekly using a bench-top set of digital scales within the CBS animal facility.

### 2.3 Cardiomyocyte isolation and transfection

Adult rat ventricular cardiomyocytes were isolated from adult male Sprague–Dawley rats (250–350 g) by a standard enzymatic technique.^21^ The ventricle was divided into thirds: apex and base were kept, discarding the mid-LV. Cardiomyocytes were cultured at 12 500 cells/well of a 24-well plate for contractility measurement. Transfection for 48 h with 100 nM pre-miR negative control #2, hsa-pre-miR-16, hsa-pre-miR-26a, anti-miR negative control #1, hsa-anti-miR-16, and hsa-anti-miR-26a was carried out as required using Lipofectamine 3000 as per the manufacturer’s protocol. miR-16 and miR-26a up-regulation were validated by PCR (Supplementary material online, *FigureS3*).

### 2.4 Cardiomyocyte contractility studies

Percentage shortening of the cardiomyocyte during contraction was measured using an IonOptix Contractility System in Krebs-Henseleit solution (37°C for rat, 32°C for human) at 1–2 mL/min and field stimulated (20–50 V, 0.5 Hz, 0.5 ms for rat, 5 ms for human). Experiments were conducted in a blinded manner.

### 2.5 Calcium fluorescent imaging

Cardiomyocyte calcium handling was evaluated in Normal Tyrode’s solution with Fluo-4 AM, and SR calcium content assessed using micropipette caffeine application as previously described.^22^

### 2.6 Pharmacological treatments in vitro

Adrenaline concentration–response curves were constructed by applying increasing concentrations of adrenaline, ranging from 3 × 10^−10^ M to 3 × 10^−6^ M, with half log-10 increments following stabilisationfrom the previous dose. β_2_AR responses were induced with 1 µM isoprenaline (non-selective βAR agonist) in the presence of 300 nM CGP-20712A (selective β_1_AR antagonist). Pertussis toxin (PTX) was added at 1.5 µg/mL for 12 h before the experiment, to inhibit Gαi.

### 2.7 In silico miR target identification and luciferase assays

For miR target identification, miRWalk 2.0 was used to predict 3′UTR (untranslated region) sequence homology by comparing 12 different miR databases.^23^ Genes located in 7≤ databases were included. Panther DB was used to stratify this list to obtain a list of proteins associated with contractility.^24,25^

Primers for luciferase assays were designed using Pimer3 to contain 5’-*Spe*I and 3’-*Hind*III restriction sites for incorporation into the pmiRReport vector. Primers were then checked in TargetScan and Ensembl to confirm the putative miR binding sites. Primers were constructed from rat cDNA using PCR and incorporated into the vector. These were expanded in *Escherichiacoli* and sequenced. Luciferase assays were performed in HEK293 cells with miR upregulation and normalized to β-galactosidase plasmid activity.

### 2.8 mCherry fluorescence intensity

Tissue sections from heart or brain were fixed in paraformaldehyde, frozen in OCT and cryosectioned. mCherry fluorescence was measured using widefield microscopy. Images were analysed using Image J with the average of three 1000 × 1000 pixel areas per image.

### 2.9 RTqPCR

RNA was isolated from cells and tissue using the Trizol protocol, and from serum using the miRNeasy Serum/Plasma isolation kit. cDNA was manufactured using specific primers for miRs with TaqMan^®^ MicroRNA Reverse Transcription Kit, and global cDNA produced for mRNA with the High-Capacity cDNA Reverse Transcription Kit. RTqPCR was carried out using a QIAgility robot for automated pipetting with specific Taqman primers for miR and mRNA targets. These were read by a Quantistudio 12K Flex 384-well plate reader and data analysed using the comparative cycle threshold method (ΔCT).^26^

### 2.10 Western blot

Protein was isolated using RIPA buffer and ran in a 17-well Bolt™ 4–12% Bis-Tris Plus Gels containing 1X Bolt™ MOPS SDS Running Buffer. Precision Plus Protein™ Dual Color Standard was used as a protein ladder and gels run for 32 min at 200 V.

Gels were transferred using an iBlot™ 2 PVDF Transfer Stack in an iBlot 2 Dry Blotting System. Antibodies were 1:200 rabbit anti-CACNA1C (Abcam, ab58552), 1:10 000 rabbit anti-vinculin (Abcam, ab129002), 1:500 rabbit anti-RGS4 (Abcam, ab9964), 1:X rabbit anti-CACNB1 (Sigma, AV34953), 1:X rabbit anti-GNB1 (ThermoFisher Scientific, PA1-725), and 1:500 rabbit anti-GNG12 (ThermoFisher Scientific, PA5-75620). Anti-rabbit HRP secondary antibody (1:2000, Cell Signalling Technologies, 7074S) was used. Membranes were developed using Clarity™ Western ECL Blotting Substrates and imaged using a ChemiDoc imaging system. Images were analysed using ImageJ with vinculin as the loading control.

### 2.11 Electrophysiology

A whole-cell patch clamp configuration was used to record calcium currents (*I*_CaL_). Pipettes (2.5–3 MΩ) contained 90 mM Cs methanesulfonate, 20 mM CsCl, 10 mM HEPES, 4 mM Mg-ATP, 0.4 mM Tris-GTP, 3 mM CaCl_2_, and 10 mM EGTA at pH 7.2 (adjusted with CsOH). Myocytes were superfused with modified Tyrode solution containing 120 mM TEACl, 10 mM CsCl, 2 mM CaCl_2_, 1 mM MgCl_2_, 10 mM HEPES, 10 mM glucose at 37°C, and pH 7.4 (adjusted with CsOH). Data acquisition and analysis were performed using pClamp software.

*I*_CaL_ was elicited by test pulses (400 ms) from a holding potential of −80 to +50 mV in 10 mV increments at 1 Hz. A conditioning pulse (−80 to −40 mV for 200 ms) inactivated residual cardiac sodium current before test pulses. *I*_CaL_ was normalized to cell capacitance. Recovery from fast inactivation was assessed with a 500 ms conditioning pulse from −80 to 0 mV was followed by a 600 ms test pulse from −80 to 0 mV in varying intervals from 0 to 600 ms in 50 ms increments with −80 mV holding potential. Current amplitude was normalized to the peak current. Steady-state inactivation curves were obtained with 500 ms conditioning pulses from a holding potential of −80 to +20 mV in 10 mV increments, followed by a 600 ms test pulse to 0 mV. Current amplitude was normalized to the peak current (*I*/*I*max).

### 2.12 Statistics

Data are represented as mean ± SEM as standard with *P* < 0.05 set for the level of significance. Mean ± SD is shown instead where *n* is greater than 30. All individual data points are shown where possible. *In vivo* AAV treatment was blinded at administration of virus, and echocardiographic data were also analysed blind by an independent imaging expert. *In vitro*, the experimenter was blinded to miR pre-treatment status of cardiomyocytes. Differences between two data sets or comparison to paired baseline was determined using Student’s t-tests, and more than two with one-way ANOVA using Tukey’s post-hoc. Repeated measures ANOVA with Bonferroni’s post-hoc test was used for time course experiments. A non-linear regression compared agonist vs. response for concentration–response curves, where an *F*-test compares the two fitted curves to test for significance. Significance is presented throughout as: *P < 0.05, **P < 0.01, ***P < 0.001.

## 3. Results

### 3.1 miR serum levels were unchanged after TTS induction

**Figure 1:**
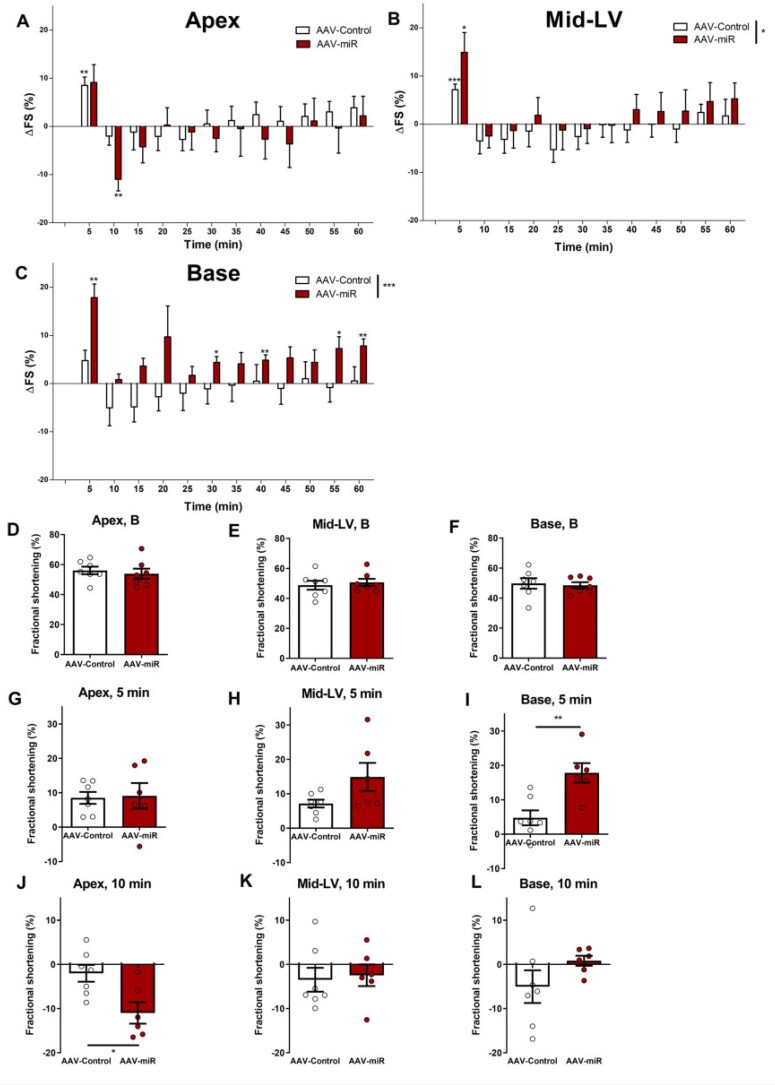
TTS was recapitulated *in vivo*. ΔFS from baseline (B), measured every 5 min for 60 min following adrenaline/saline (*A*-apex, *B*-mid-LV, *C*-base). Serum miR-16 (*D*) and miR-26a (*E*) at B, 20 and 60 min post-adrenaline/saline (normalised to B, *N* = 6). Mean ± SEM shown, significance by repeated measures (RM)-ANOVA, and Student’s *t*-test comparing timepoints to B. *P < 0.05, **P < 0.01, ***P < 0.001.

The catecholamine-induced TTS model^12^ delivers 55 µg/kg adrenaline via the external jugular vein of an anaesthetised rat (maintained on 1.5–2.5% isoflurane in 1.5 L/min 100% O_2_ as described in Section 2.1), in order to focus on immediate responses (within 1 h). Contractility at apex, mid-LV, and base (*Figure 1A–C*) were measured as change in FS from baseline every 5 min after adrenaline delivery. Contractility was significantly increased at 5 min throughout the heart, which represents the expected positive inotropic effect of adrenaline. After 15 min, there was a profound reduction in apical contractility which was significant between 15 and 45 min and recovered between 50 and 60 min. In the mid-LV, the negative inotropy was brief, being significant at 15, 20, and 30 min, and recovered to baseline from 40 min. For the base, the reduction was significant at 15 min, recovered from 20 min, and trended towards hypercontractility from 45 min. These spatial differences reproduced the characteristic apical/basal pattern of TTS.

miRs were isolated from blood at baseline, 20 min, and 60 min. Both miRs were unchanged vs. baseline/saline within an hour post-adrenaline (*Figure 1D and E*). Therefore, an adrenaline dose sufficient to induce TTS did not itself stimulate miR-16 or miR-26a production/release.

### 3.2 AAV-miR in vivo sensitised to TTS

AAV co-expressing miR-16 and miR-26a were injected into rat tail veins, since these miRs were elevated together *in vivo*. Both control (AAV-control) and miR (AAV-miR) vectors contained a mCherry reporter for infection assessment. By 6 weeks, AAV-control and AAV-miR hearts had significantly higher mCherry fluorescence than control untreated rat tissue (Supplementary material online, *FigureS1A* and *C*), with no apex/base difference. However, it was not possible to detect tissue miR increases by PCR at the single 6-week timepoint chosen.

**Figure 2:**
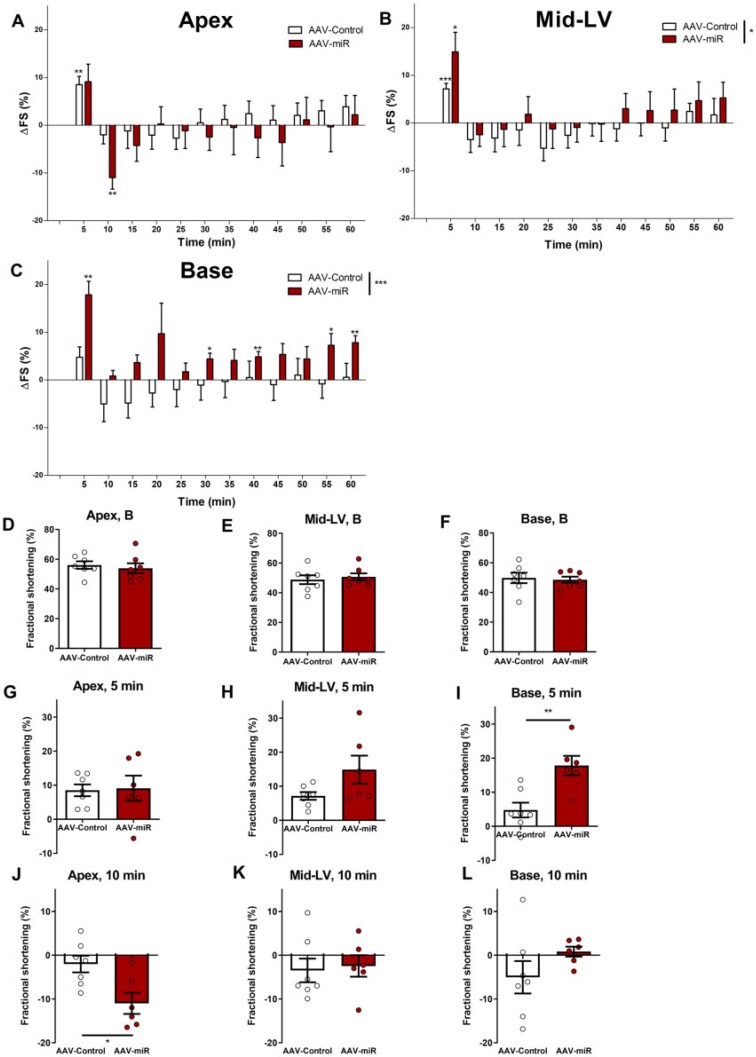
AAV-miR produces TTS-like contractility *in vivo* following adrenaline. Six weeks after AAV-miR and AAV-control. Overall time-course are shown (*A*-apex, *B*-mid-LV, *C*-base). FS shown at baseline (*D*-apex, *E*-mid-LV, *F*-base), ΔFS at 5 min (*G*-apex, *H***-**mid-LV, *I*-base), and ΔFS at 10 min post-adrenaline (*J***-**apex, *K*-mid-LV, *L***-**base). Mean ± SEM shown. For *A–C* and *G–L, N* = 7 for AAV-control and *N* = 6 for AAV-miR. For *D–F, N* = 7 throughout. Significance for *A–C* RM-ANOVA (displayed in figure key), and Student’s *t*-test comparing each timepoint to baseline and *D–L* by Student’s *t*-test. *P < 0.05, **P < 0.01, ***P < 0.001.

Baseline FS was unchanged at apex, mid-LV, or base in AAV-miR hearts *in vivo* (*Figure 2D–F*). Pilot studies showed increased adrenaline sensitivity and therefore this experiment utiliseda lower adrenaline dose *versusFigure 1*(18 vs. 55 µg/kg adrenaline). Adrenaline-induced changes at this lower concentration were less marked than with the higher dose in AAV-control (cf. *Figure 1*). However, AAV-miR developed TTS-like changes even at this more modest dose (*Figure 2A–C*). The overall time-course was significantly different in mid-LV (*P* < 0.05) and base (*P* < 0.001) between AAV-miR and AAV-control.

Key timepoints were directly compared: baseline (*Figure 2D–F*), 5 min (*Figure 2G–I*), and 10 min (*Figure 2J–K*) post-adrenaline. The initial positive response at 5 min was unchanged in the apex (*Figure 2G*) but was significantly increased by AAV-miR in the base (*Figure 2I*) and trended towards an increase in mid-LV. Adrenergic response at the base was significantly increased in AAV-miR (at 5, 30, 40, 55, and 60 min) but not in AAV-control (*Figure 2C*). This implied that AAV-miR sensitisedbasal myocardium to adrenaline-mediated inotropy. In contrast, apical contractility 10 min post-adrenaline exhibited a significantly greater reduction after AAV-miR treatment than AAV-control (*Figure 2J*), with mid-LV or base being unchanged (*Figure 2K and L*). Therefore, AAV-miR accentuated the apex/base difference to high-dose adrenaline and reduced the threshold for developing TTS.

We evaluated rat activity and growth rates because of the potential neuropsychiatric association of TTS, miR-16 and miR-26a.^3,17–19^ Activity was increased and animal weight reduced over the 6 weeks by AAV-miR treatment (Supplementary material online, *FigureS2*). No signs of distress/pain were detected by the Rat Grimace Scale throughout. There was a significantincrease in mCherry expression in AAV-Control treated rat brains at 6 weeks, and a trend to increase in AAV-miR treated rat brains (*P* = 0.1294, Supplementary material online, *FigureS1B*).

### 3.3 miRs-reduced baseline contractility in apical but not basal isolated cardiomyocytes

**Figure 3:**
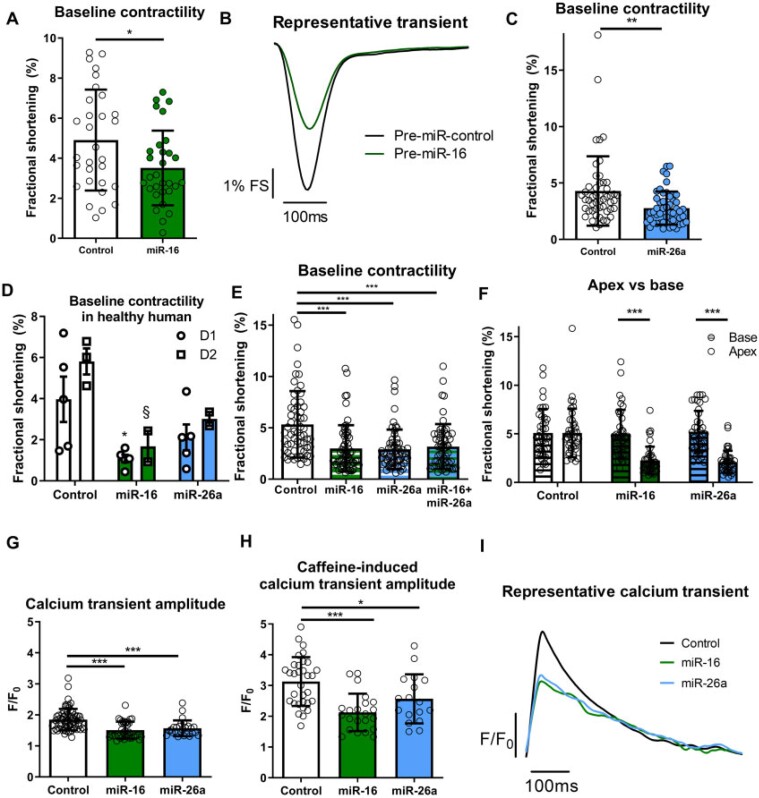
miRs reduce baseline contractility and calcium transient amplitude of apical cardiomyocytes. Adult rat apical cardiomyocytes FS shown with miR-16 [*A, n*/*N* = 30/6, representative contractile transient (*B*)] and miR-26a (*C, n*/*N* = 50/10). (*D*) Baseline FS of healthy human adult apical cardiomyocytes with TTS-miRs shown from donor 1 (D1, control = 5, miR-16 = 5, and miR-26a = 5, *P < 0.05 vs. control) and donor 2 (D2, control = 3, miR-16 = 2, and miR-26a = 2, §*P* < 0.05 vs. control) with one-way ANOVA for each heart separately. (*E*) Co-transfection of TTS-miRs (*n*/*N* = 60/6) in apical rat cardiomyocytes. (*F*) Paired apical/basal adult rat cardiomyocytes with TTS-miRs (*n*/*N* = 40/4). (*G*) Calcium transient amplitudes (control = 66/4, miR-16 = 37/4, and miR-26a = 26/4). (*H*) Caffeine-induced calcium transient amplitudes (control = 32/4, miR-26 = 22/4, and miR-26a = 17/4). (*I*) Representative calcium transient (Δamplitude over time). ‘*n*’ = cardiomyocytes and ‘*N*’ = rats. Mean ± SEM shown for *D*, Mean ± SD shown for *A, C, E, F, H*, and *I*. Significance was determined by Student’s *t*-test for *B* and *C*. For *C*, the cells in the control arm with 14% and 18% FS may be considered outliers, but exclusion did not affect the level of significance. For *D, E, G*, and *H*, significance was determined by one-way ANOVA with Tukey’s post-hoc and two-way ANOVA with Bonferroni post-hoc for *F*. *P < 0.05, **P < 0.01, ***P < 0.001.

**Figure 4:**
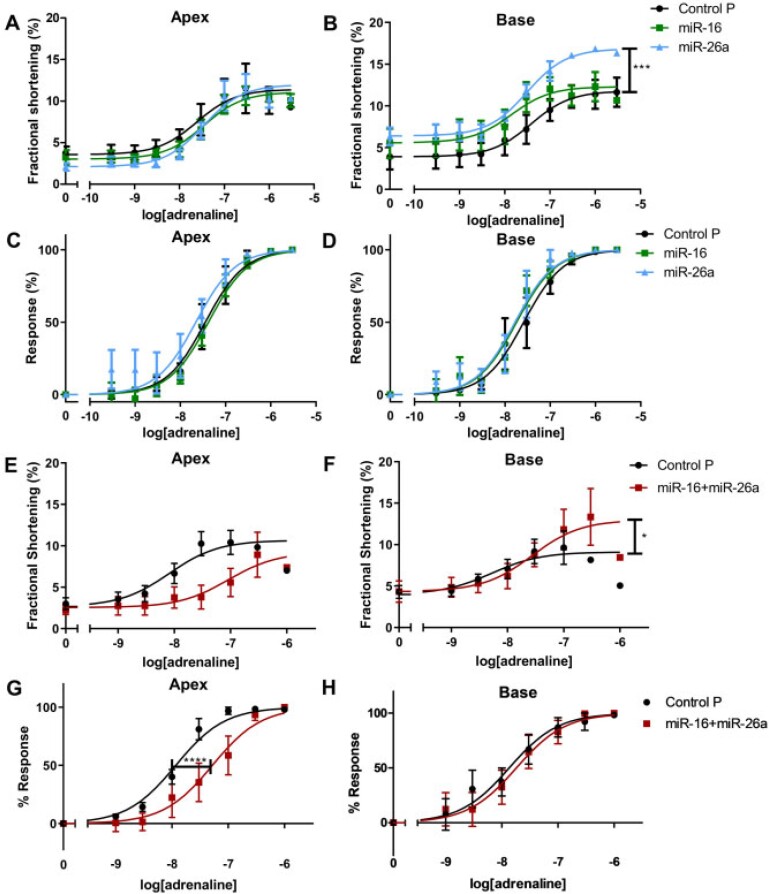
TTS-miRs reduce apical adrenergic sensitivity and increase basal maximum adrenergic response. TTS-miR adrenaline concentration-response curves in apical/basal cardiomyocytes. (*A***/***B*) FS in apex/base. (*C***/***D*) Normalisedresponse in apex/base. (*E/F*) FS in apex/base with TTS-miRs together. (*G/H*) Normalisedresponse in apex/base with TTS-miRs together. *n*/*N* = 6/6. Mean ± SEM shown, *n*’ = cardiomyocytes and ‘*N*’ = rats, significance by non-linear regression comparing agonist vs. response for *A–H* (*F*-test, Supplementary material online, *Table* *S1*).

**Figure 5:**
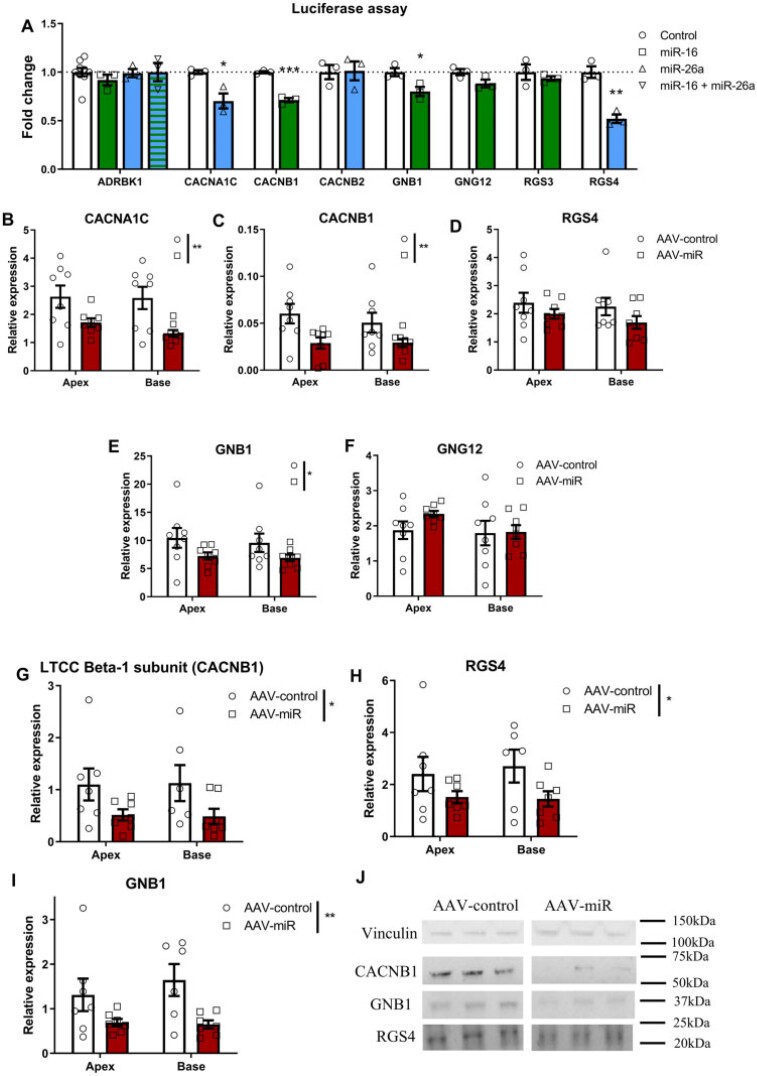
AAV-miR-reduced CACNB1, RGS4, and GNB1. (*A*) Luciferase plasmid activity in HEK293 cells with TTS-miRs for ADRBK1, CACNA1C, CACNB1, CACNB2, GNB1, GNG12, RGS3, and RGS4. *n*/*N* = 9/3, ‘*n*’ = transfections and ‘*N*’ = biological repeats, significance with ‘*N*’ by Student’s unpaired *t*-test. RTqPCR of CACNA1C (*B, N* = 8), CACNB1 (*C, N* = 8), RGS4 (*D, N* = 8), GNB1 (*E, N* = 8), and GNG12 (*F, N* = 8) from apex/base of AAV-treated rat hearts. Western blot for CACNB1 (*G*), RGS4 (*H*), and GNB1 (*I*) from apex/base of AAV-treated rat myocardium with representative blots and molecular weights shown (kDa, *J*). For *G–I, N* = 7 for AAV-control and AAV-miR in apex and AAV-miR in base, and *N* = 6 for AAV-control in base. Mean ± SEM displayed throughout. For *B–I* ‘*N*’=number of rats. Significance by two-way ANOVA, with Bonferroni’s post hoc: *P < 0.05, **P < 0.01, ***P < 0.001.

*In vitro* transfection of pre-/anti-miRs (to increase and reduce miRs, respectively) was performed in isolated adult rat cardiomyocytes (Supplementary material online, *FigureS3*). Increased miR-16 and miR-26a significantly and markedly reduced baseline contraction of apical cardiomyocytes (where mean ± SD fractional shortening is 4.9 ± 2.5% for control vs. 3.5 ± 1.9% for miR-16, and 4.3 ± 3.1% for control vs. 2.8 ± 1.5% for miR-26a, *Figure 3A–C*), although anti-miR had no effect (Supplementary material online, *FigureS4*). Contraction kinetics (Supplementary material online, *FigureS5*) and proportion of cardiomyocytes beating (not shown) were unchanged. As TTS-miRs are concomitantly increased *in vivo*, co-transfection was conducted, but produced no additional reduction (*Figure 3E*). TTS-miR effect was compared between paired apical and basal cardiomyocytes. Contractility in basal cardiomyocytes was unchanged, whereas apical cell contractility was reduced vs. control and basal cardiomyocytes for both miRs (*Figure 3F*).

### 3.4 TTS-miRs reduced contractility in healthy human cardiomyocytes

Two human hearts from normal donors (one male, 39 years, with resuscitated cardiac arrest, rejected for transplant due to increased lactate and K^+^, and one female, 32 years, with a brain haemorrhage, rejected for transplant due to travel time >6 h) were utilisedto study the biological effect of TTS-miRs in healthy human cardiomyocytes. In primary human adult apical cardiomyocytes from these hearts, miR-16 pre-treatment significantly reduced baseline contractility (*Figure 3D*), as in rat apical cardiomyocytes, and miR-26a trended towards a reduction.

### 3.5 miRs impaired calcium cycling

**Figure 6:**
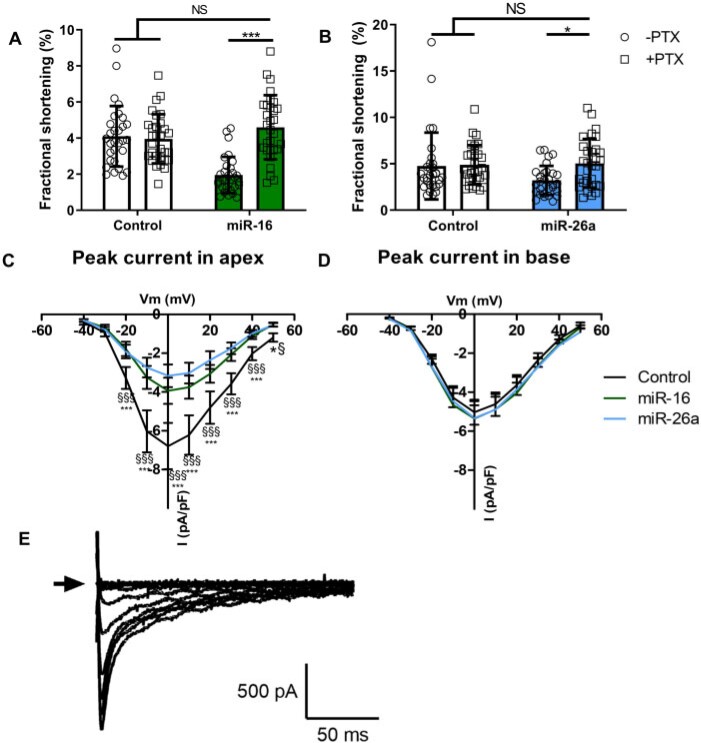
TTS-miRs altered Gαi and calcium current. Baseline FS of miR-16 (*A, n*/*N* = 30/6) and miR-26a (*B, n*/*N* = 30/6) transfected adult rat apical cardiomyocytes pre-treated with PTX. (*C/D*) Peak calcium current amplitude in apex (control = 11/7, miR-16 = 11/7, and miR-26a = 11/6) or base (control = 16/8, miR-16 = 14/7, and miR-26a = 10/6). (*E*) Representative patch clamp protocol. ‘*n*’ = cardiomyocytes and ‘*N*’ = rats. Mean ± SD shown for *A* and *B*, Mean ± SEM shown for *C* and *D*, significance by two-way ANOVA, with Bonferroni’s post hoc (*A* and *B*: *P < 0.05, **P < 0.01, ***P < 0.001) and RM-ANOVA (*C* and *D*: *P < 0.05 for miR-16 vs. control, ^§^*P* < 0.05 for miR-26a vs. control). For *B*, the cells in the control arm with 14% and 18% FS may be considered outliers, but exclusion did not affect the level of significance.

Both miRs reduced calcium transient amplitude (*Figure 3G*), but time to peak and rate of calcium decay were unchanged (Supplementary material online, *FigureS6*), as represented in *Figure 3I*. TTS-miRs significantly reduced caffeine-induced calcium transient amplitude, which illustrates a reduced sarcoplasmic reticulum (SR) calcium content (*Figure 3H*), but fractional release was unaltered (Supplementary material online, *FigureS6*). Caffeine-induced calcium transient decay showed SERCA (SR Ca^2+^-ATPase) and NCX (Na^+^/Ca^2+^ exchanger)-mediated contributions were unchanged, although there was a minor effect on slow mechanisms of calcium decay (Supplementary material online, *FigureS6*).

### 3.6 miRs differentially altered apical-basal adrenergic response in rat isolated cardiomyocytes

Apical cardiomyocyte adrenaline concentration–response curves (β_1_AR + β_2_AR stimulation) were unchanged by either miR alone (absolute or normalised, *Figure 4A and C*, Supplementary material online, *TableS1A* and *B*). The maximal amplitude of basal cardiomyocytes was significantly increased with miR-26a (*P* < 0.001, *Figure 4B*), with differences removed after normalisationto maximum amplitude (*Figure 4D*). In combination, miR-16 and miR-26a also significantly increased maximum amplitude of basal (but not apical) cardiomyocytes (*Figure 4E* and *F, P* < 0.05). The sensitivity (EC50) of basal cardiomyocytes was unchanged with combined miRs (*Figure 4H*). In contrast, this combination decreased apical sensitivity to adrenaline (*Figure*4G, P < 0.0001). The effect of stimulation of β_2_AR alone (adrenaline plus β_1_AR-blocker) was unchanged with either miR, as was peak contractility or change from baseline (Supplementary material online, *FigureS7*).

### 3.7 Luciferase assay validates miR targets

*In silico* TTS-miR target analysis was conducted to identify potential mechanism(s). miRWalk 2.0 was used to predict 3′UTR sequence homology by comparing 12 different miR databases.^23^ Genes located in 7 ≤ databases were included. Panther DB was used to stratify this list to obtain a list of proteins associated with contractility.^24,25^

Considering miRs silence/degrade mRNA and subsequently reduce protein expression, we chose protein targets that could explain the changes in contractility, adrenergic system, and calcium handling that we observed when reduced. We excluded the β_2_AR because of the lack of change of the response after miR treatment (Supplementary material online, *FigureS7*) and adenylyl cyclase since, in our hands, reduction of cAMP is a minor part of the negative inotropic effect of Gαi.^12^ Only binding sequences conserved in humans were included, which yielded 33 miR-16 targets, and 29 for miR-26a (Supplementary material online, *TableS2A* and *B*). Targets chosen for miR-16 were CACNB1, GNB1, GNG12, and RGS3, and miR-26a as CACNA1C, CACNB2, and RGS4. ADRBK1 was included as it is required for stimulus trafficking.

Luciferase assays showed miR-16 reduced CACNB1 and GNB1 expression, and trended to reduce GNG12, but not ADRBK1 or RGS3. miR-26a reduced CACNA1C and RGS4 expression, but not ADRBK1 and CACNB2. Both miRs target ADRBK1 but co-transfection had no effect (*Figure 5A*).

### 3.8. *In vivo* AAV-miR reduced CACNB1, RGS4, and GNB1

RTqPCR evaluated expression in apex and base *in vivo* after AAV-miR, where CACNA1C, CACNB1, and GNB1 expression were significantly reduced (*Figure*5B, C, and *E*), although RGS4 and GNG12 were not (*Figure 5D and F*). GNG12 was omitted further as luciferase assay and RTqPCR were unchanged. Western blots showed CACNB1, RGS4, and GNB1 protein to be reduced by AAV-miR, with no apex/base difference (*Figure 5G–I*), with representative unedited cropped blots seen in *Figure 5J*.

### 3.9 Pertussis toxin pre-treatment abolished TTS-miR negative inotropy

Since GNB1 and RGS4 both regulate Gαi, we inhibited Gαi with pertussis toxin (PTX) *in vitro*. This prevented the reduction in baseline contractility in apical cardiomyocytes for both miRs with control unchanged (*Figure 6A and B*).

### 3.10 miRs-reduced calcium current

As CACNB1 was reduced, we investigated L-type calcium channel (LTCC) function using patch clamp. Apical (but not basal) cardiomyocyte peak LTCC current was significantly reduced by both miRs (*Figure 6C and D*). Inactivation and recovery curves for apex and base were unchanged with either miR (Supplementary material online, *FigureS8*). Representative patch clamp traces can be seen in *Figure 6E*.

## 4. Discussion

TTS represents a poorly understood acute cardiac illness with significant mortality and long-term morbidity.^3,7,8^ TTS pathogenesis and the specific role for miR-16 and miR-26a (acutely elevated in TTS patients) are unknown. Serum miRs were unchanged acutely by adrenaline in our rat model of TTS *in vivo*, showing that their elevation is not induced as a transient response to the catecholamine surge. We therefore suggest that their increase in TTS patients may have predated the catecholamine rise that caused the acute episode and could predispose to and/or exacerbate the TTS-like phenotype. Here, we have demonstrated multiple effects of TTS-miRs *in vivo* and *in vitro* consistent with this hypothesis.

Cardiac AAV-miR co-overexpression *in vivo* accentuated ventricular apical hypokinesis and basal hypercontractility following adrenaline challenge. This gives a novel insight to the syndrome, suggesting that both basal and apical responses are amplified in TTS and that the apicobasal gradient is accentuated with TTS-miRs. We previously showed the initial magnitude of positive adrenergic response is important for PKA-dependence of β_2_AR phosphorylation to switch from Gαs (stimulatory) to Gαi (cardiodepressive/protective).^12^ In rat myocardium overexpressing TTS-miRs there is enhanced initial sensitivity to adrenaline and therefore greater propensity for Gαs-Gαi switch. The mechanisms underlying this, and the apicobasal differences were explored in detail in tissue from the treated hearts and in isolated cardiomyocytes from untreated animals.

Apical, but not basal, cardiomyocytes demonstrated a dramatic decrease in contractility after pre-treatment with either pre-miR-16 or pre-miR-26a. This finding was confirmed in non-failing human cardiomyocytes. There was no further decrease for the rat apical cardiomyocytes when the miRs were combined, implying unified action or maximal effect at this concentration. The absent reciprocal changes with anti-miRs (Supplementary material online, *FigureS4*) suggest miR-16 and miR-26a do not regulate contractility in resting cardiomyocyte physiology but rather affect the system when raised in disease. It is interesting that miRs reduced baseline contractility *in vitro* but not *in vivo* [though note a minor non-significant reduction in apex (*Figure 2D*)], possibly due to higher *in vitro* miR concentrations, considerable timescale differences and physiological whole organism compensation *in vivo* masking resting differences.

Furthermore, there were apicobasal miR differences in adrenergic response *in vitro* (*Figure 4*). Adrenaline response in basal cardiomyocytes was significantly enhanced by miR pre-treatment without a shift in sensitivity, with miR-26a predominating in the effect. Apical cardiomyocytes required both miRs to reveal the reduced sensitivity to adrenaline, providing a cellular basis for the reduced *in vivo* adrenergic response. Overall, synergism of TTS-miRs appears necessary to fully reproduce the TTS phenotype post-adrenaline.

The reduced calcium transient amplitude with TTS-miRs (*Figure 3G*) would provide an explanation for decreased cardiomyocyte contractility, but whether this explains the magnitude of change is unclear since the calcium–force relationship is non-linear. We observed reduced SR content with unchanged fractional release, suggesting RyR open probability (*P*_o_) is likely not altered.^27^ The reduced SR content was not a result of impaired SR reuptake, since SERCA function was unchanged. Similarly, there was no change in the rate of NCX-mediated calcium extrusion. The altered rate of slow mechanisms of calcium extrusion with miR-26a is likely unimportant since these are not thought to regulate beat-to-beat contraction. SR calcium reduction is thus a probable consequence of reduced cellular calcium secondary to reduced LTCC current (*Figure 6C*), which could result from the observed downregulation in CACNB1 [encoding LTCC beta-1 subunit (Ca_v_β)]. LTCC current is markedly reduced if Ca_v_β is absent, and reintroduction enhances surface expression and increases *P*_o_ by shifting activation to more hyperpolarized voltages.^28^

miRs produced TTS-like changes in adrenergic response *in vitro* which could be explained by the observed reductions in GNB1 and RGS4. GNB1 represents G-protein subunit beta-1 (Gβ), which dimerises with Gγ to bind Gαs/Gαi when inactive, constituting the heterotrimeric G-protein. Upon G-protein-coupled receptor (GPCR) activation, Gα-Gβγ dissociate to exert downstream effects. Gαs liberation causes inotropy and lusitropy. Gβγ can independently activate GPCR kinases (GRKs), which terminate Gαs signalling by receptor internalisationand stimulus trafficking to β_2_AR-Gαi.^29^

RGS4 (regulator of G-protein signalling 4) accelerates Gα-GTPase activity (<2000-fold) to terminate Gαi and Gβγ signalling and antagonises Gα-mediated signal generation.^30^ Therefore, reduced RGS4 would strongly potentiate Gαi. Additionally, we previously showed that the initial adrenergic cAMP response drives PKA to phosphorylate β_2_AR and produce the Gαi switch.^12^ Therefore, increased activation of Gαs can indirectly produce decreased contractility in the vulnerable apex through greater Gαi coupling. The two TTS-miRs acted synergistically to profoundly reduce the apical positive inotropic effect produced byadrenaline (*Figure 4G*). miR-16 targets Gβ and miR-26a targets RGS4, suggesting multiple points of action within this pathway. The action of PTX (which inactivates Gαi) to prevent the negative inotropy (*Figure 6A and B*) supports these miRs affecting contraction through Gαi. The impact of reductions in GNB1 and RGS4 on the adrenergic system is complex, but reproduce key differences in apical–basal adrenergic responses as in TTS and provide important mechanistic insight into TTS-miR action.

Surprisingly, maximum β_2_AR responses were unaffected by TTS-miRs (Supplementary material online, *FigureS7*). We previously demonstrated the importance of β_2_AR-Gαi stimulus trafficking in causing the regional apical reduction in contractility in TTS.^12^*In vitro* culture may have affected β_2_AR-Gαi dependence since RGS2, which terminates β_2_AR-Gαi signalling, increases in the absence of agonist stimulation in culture.^31^

### 4.1 How are apical-basal differences explained?

The apex/base divergence shown here *in vivo* and *in vitro* with TTS-miRs reflects those in patients with TTS. The striking difference in baseline contractility between apical and basal cardiomyocytes after miR treatment (*Figure 3F*) illustrated that these inherent differences alone could underlie the spatial changes in TTS without invoking further mechanisms. However, these functional alterations occur in the rat despite no difference between apex and base in the protein changes (CACNB1, RGS4, and GNB1) caused by TTS-miRs. This is perhaps to be expected, since miRs exert their biological affect on protein expression regardless of cellular location. Factors that cause the functional divergence could include inherent differences in apical-basal adrenergic components,^32^ such as the greater apical β_2_AR density and β_2_AR-Gαi response,^12,32^ thus favouring the negative inotropic signalling axis.^12^ Subcellular compartmentation may also play a role and we recently demonstrated differences between apex and base in this regard.^32^

Alongside our data which show that TTS-miRs predispose to TTS by altering adrenergic signalling and calcium handling, recent evidence shows further mechanisms of involvement of βAR signalling in TTS. Increased GRK2 and β-arrestin2 have been identified in TTS patients,^33^ and it is known that GRK2 and β-arrestin recruitment is necessary for β_2_AR-Gαi stimulus trafficking.^29^ Additionally, a recent TTS-iPSC-CM patient line showed increased β_1_AR, β_2_AR and cAMP responses, increased calcium transient amplitude and kinetics, and higher sensitivity to catecholamines with reduced 24 h desensitization of TTS-iPSC-CMs EHTs.^34^ This is in line with our observation that increased sensitivity to the positive inotropic effects of adrenaline is an integral part of the TTS syndrome.

### 4.2 Use of AAV9 for cardiac infection in rat *in vivo*

AAV9 was selected as this has been shown to be the most efficient AAV serotype for infection of cardiomyocytes within the heart.^35^ Indeed, AAV9 can effectively cross endothelial barriers to reach the heart.^36–38^ Similarly, AAV9 can cross the blood–brain barrier,^39^ and this was desirable, since although the origin of circulating miR-16 and miR-26a is unknown, Jaguszewski *et al*.^15^ proposed they were from neuronal tissue. Since miR-16 and miR-26a are associated with neuropsychiatric disorders^17–19^ that are frequent in TTS,^3^ we combined AAV9 with a generalized EF1a promoter to obtain upregulation of miRs in other organs, including the central nervous system. We noted a significant increase in mCherry expression in both the heart and the brain (Supplementary material online, *FigureS1*), demonstrating that AAV9 was able to infect both tissues.

### 4.3 TTS-miRs and the brain-heart interaction in TTS

Alterations in brain structure/function is now being understood in TTS, with changes in the ANS and limbic system.^10,11^ Our pilot observations showed that TTS-miRs altered animal behaviour (Supplementary material online, *FigureS2*) in a way consistent with increased anxiety, but robust behavioural analysis is required to comment further. This, along with the known associations of miR-16 and miR-26a^17–19^ and TTS with neuropsychiatric disorders,^3^ raises the possibility that miR-16 and miR-26a are chronically raised in situations of ongoing stress, and given our findings, could represent a priming factor to predispose to an increased likelihood of future TTS. miR-16 and miR-26a thus represent novel effectors in the brain–heart interaction within TTS.

### 4.4 Future work

A chronic stress model is ideally required to understand the link between prior stress, the miR-16 and miR-26a increase and TTS development over a longer time window. However, an animal model of chronic psychological stress is challenging from ethical and regulatory standpoints. Additionally, both the source of the miRs and range of final cellular targets need to be completely defined. We have used systemic administration of AAV miRs *in vivo*, since there is no specific reason to believe that release of miR-16 and miR-26a is from the heart itself. In terms of targets, we have limited the scope of our investigation to the effect of TTS-miRs on the contractility in cardiomyocytes, as the primary acute symptoms are from regional myocardial hyper- and hypo-contractility. However, vascular changes have also been implicated in TTS, so effects of miRs on adrenergic sensitivity in blood vessels is also important to investigate, as well as longer-term changes using models where catecholamine exposure is sustained.

## 5. Conclusion

TTS-miRs are not bystander markers of acute adrenaline release but predispose to TTS. Since they are raised in stress, anxiety, and depression, they could be part of a priming mechanism where chronic stress (known to be a factor in TTS patients) predisposes to an acute episode.

## Supplementary material

Supplementary material is available at *Cardiovascular Research* online.

## Author contributions

L.S.C. completed the main body of *in vivo* and *in vitro* work, supervised the contribution of others, and has written this work. J.F. supervised and assisted with design and expertise for bioinformatic identification/confirmation of miR targets. G.C., R.C., L.M.W., L.F., and A.A.D. contributed to *in vitro* contractility experiments. E.D. conducted the electrophysiological experiments. J.F., G.C., and B.X.W. conducted western blots. P.P. and G.C. conducted immunofluorescent imaging. R.J. and P.W. assisted with *in vivo* work. M.S. acted as an expert echocardiographer and independently analysed the *in vivo* data in a blinded fashion. P.W., A.R.L., C.M.T., and T.T. were involved in supervisory capacities. S.E.H. contributed to the main supervision of this work, obtained funding, designed this project along with T.T., and has written this work.

## Funding

This work was supported by a British Heart Foundation MBPhD studentship for LSC supervised by SEH (grant number FS/16/52/32259). T.T. was supported by DFG (KFO311).

**Conflict of interest:** T.T. filed and licensed patents about non-coding RNAs. T.T. is a founder and shareholder of Cardior Pharmaceuticals GmbH.

## Data availability

The data underlying this article will be shared on reasonable request to the corresponding author.

**Translational perspective**

Takotsubo syndrome (TTS)-associated miRs have the potential to be active players predisposing to TTS. Feasibly, their measurement in recovered TTS patients during subsequent periods of stress could be used to predict likelihood of recurrence, a significant risk in this population, and allow preventative action. Since they have been reported as raised in anxiety and depression, they could represent a priming mechanism where chronic stress predisposes to an acute episode. Understanding the mechanistic basis for the sensitisationmay also allow design of other prophylactic pharmacological therapies, including the pre-/anti-miR constructs which are now starting to reach the clinic.

## Supplementary Material

cvab210_Supplementary_Data

## Notes

This manuscript was handled by Consulting Editor Evangelia G. Kranias.
