# Supplementary material for: Circulating microRNAs predispose to takotsubo syndrome following high-dose adrenaline exposure
Source: Cardiovasc Res. 2021 Jun 22;118(7):1758–70. doi: 10.1093/cvr/cvab210 (PMC9214785; doi:10.1093/cvr/cvab210)
Supplement: cvab210_Supplementary_Data [file cvab210_supplementary_data.docx]

Supplementary Material

Circulating microRNAs predispose to takotsubo syndrome following high-dose adrenaline exposure

Short title: Circulating microRNAs predispose to takotsubo syndrome

**Authors:**

Liam S. Couch*^1,2^, Jan Fiedler^2,3^, Giles Chick^1^, Rory Clayton^1^, Eef Dries^1^, Laura M. Wienecke^1,2.4^, Lu Fu^1,5^, Jerome Fourre^1^, Pragati Pandey^1^, Anselm A. Derda^1,2,4^, Brian X. Wang^1^, Richard Jabbour^1^, Mayooran Shanmuganathan^1^, Peter Wright^1,6^, Alexander R. Lyon^1,7^, Cesare M. Terracciano^1^, Thomas Thum^1,2,3^, Sian E. Harding^1^

**Affiliations:**

^1^ National Heart and Lung Institute, Imperial College London, UK

^2^ Institute of Molecular and Translational Therapeutic Strategies, Hannover Medical School, Germany

^3^ Fraunhofer Institute for Toxicology and Experimental Medicine (ITEM), Hannover, Germany

^4^ Department of Cardiology and Angiology, Hannover Medical School, Germany

^5^ Department of Physiology, Xuzhou Medical University, China

^6^ Department of Life Sciences, University of Roehampton, London, UK

^7^ Department of Cardiology, Royal Brompton Hospital, London, UK

**Materials & Correspondence**

Dr Liam S Couch, 4^th^ Floor ICTEM, Hammersmith Hospital, DuCane Road, London, W12 0NN. Telephone: +442075943009. Email: [liam.couch11@imperial.ac.uk](mailto:liam.couch11@imperial.ac.uk)

| 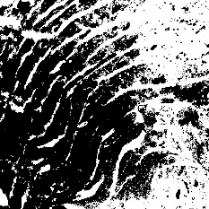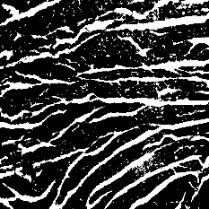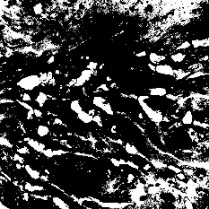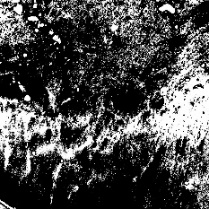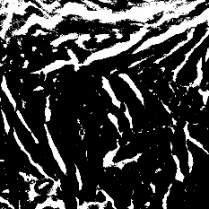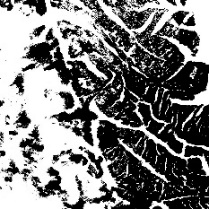 Base 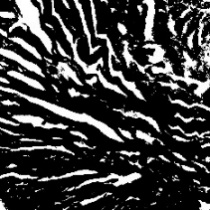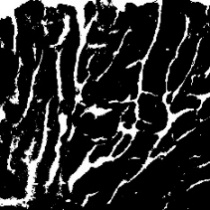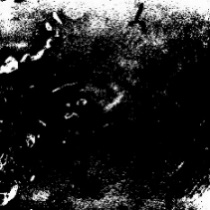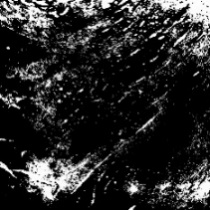 Base  AAV-miR  Apex 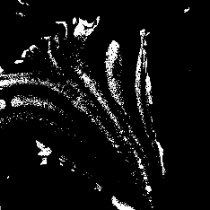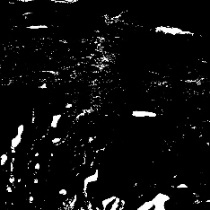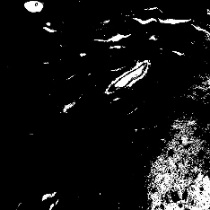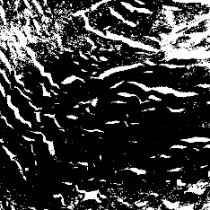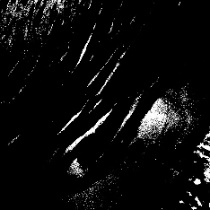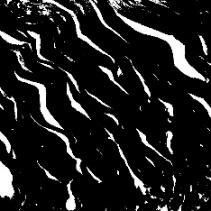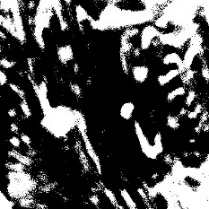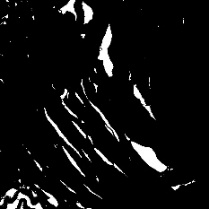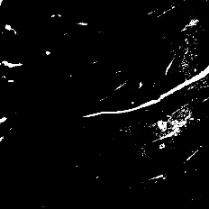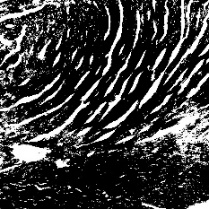 Apex  AAV-Control 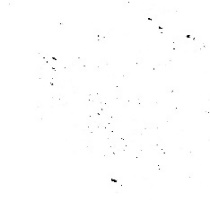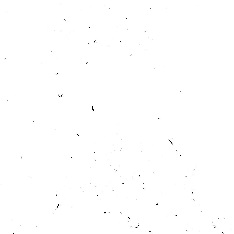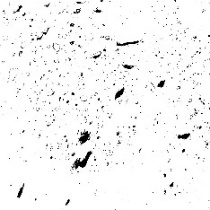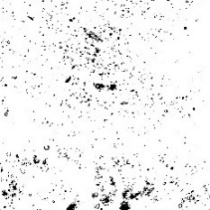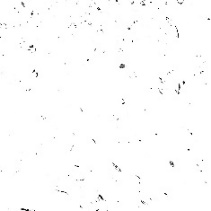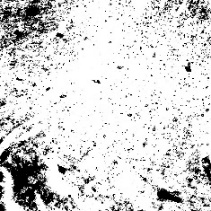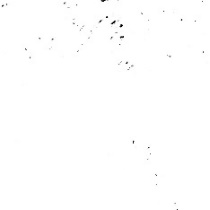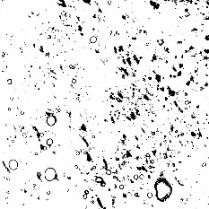 7  2  3  4  5  6  1  7  2  3  4  5  6  1  150µm  150µm  150µm  150µm  150µm  150µm  150µm  150µm  150µm  150µm  150µm  150µm  150µm  150µm  150µm  150µm  150µm  150µm  150µm  150µm  150µm  150µm  150µm  150µm  150µm  150µm  150µm  150µm  **1A**  **C**  **B** |
| --- |

**Online Figure 1.** mCherry is increased after AAV infection.

Mean fluorescent intensity of heart and brain sections from AAV-control and AAV-miR treated rats as measured via widefield fluorescent microscopy. **A** Quantification of mean fluorescent intensity of AAV-control and AAV-miR treated rat heart tissue minus from an averaged value for untreated rat tissue (N=7 for AAV-control apex and base, and AAV-miR base, and N=6 for AAV-miR apex). **B** Quantification of mean fluorescent intensity of AAV-control and AAV-miR treated rat brain tissue minus from an averaged value for untreated rat tissue (N=10). **C** Representative images of mCherry fluorescent intensity of AAV-control and AAV-miR heart sections from treated animals at apex and base where black represents an increase in mCherry fluorescence. Data is displayed as mean ± SEM, with significance determined by comparing each condition with change from untreated rat tissue by t-test, and comparison between AAV-miR and AAV-control carried out by two-way ANOVA. Significance is shown as follows: *=P<0.05, **=P<0.01, ***=P<0.001.

|  **2A**  **B**  |
| --- |

**Online Figure 2.** AAV-miR treated animals had increased activity and reduced weight.

**A** Activity levels of AAV-control and AAV-miR treated animals. n/N=37/3 ‘n’ corresponds to the number of observed days and ‘N’ equals the number of cages observed that contained between 2 and 5 rats. **B** Weight of AAV-control and AAV-miR treated rats measured weekly (N=10). Data shown as median ± CI for **A** and mean ± SEM for **B**. Significance was determined for **A** using Mann-Whitney U test, and was significant whether based on ‘n’, the number of observed days, or ‘N’ the number of cages. Statistical differences from **B** were compared using repeated measures ANOVA comparing AAV-control and AAV-miR, as indicated in the figure legend. Significance is shown as follows: *=P<0.05, ***=P<0.001.

| **3A**  **B**  **C**  **D**  |
| --- |

**Online Figure 3.** miR-16 and miR-26a can be upregulated and downregulated in cardiomyocytes *in vitro.*

Relative expression of miR-16 and miR-26a modulated by *in vitro* transfection in adult rat apical cardiomyocytes. **A** miR-16 upregulation (N=4). **B** miR-16 downregulation (N=3 for Control A and N=4 for Anti-miR-16). **C** miR-26a upregulation (N=3). **D** miR-26a downregulation (N=3). N represents the number of transfections. Data are displayed as mean ± SEM, with significance determined by Student’s t-test and shown as follows: #=P=0.055, *=P<0.05, **=P<0.01. Control P refers to pre-miR negative control #2 and Control A refers to anti-miR negative control #1.

|  **4A**  **B** |
| --- |

**Online Figure 4.** anti-miR-16 and anti-miR-26a do not alter baseline contractility of apical cardiomyocytes.

Baseline percentage FS of adult rat apical cardiomyocytes shown with downregulation of miR-16 (**A**, n/N=30/6) and miR-26a (**D**, n/N=50/10). Numbers are displayed as n/N, where ‘n’ is equal to the number of cardiomyocytes and ‘N’ represents number of rats. Data are displayed as mean ± SEM, with significance determined using Student’s t-test.

|  **5A**  **B**  **C** |
| --- |

**Online Figure 5.** miR-16 and miR-26a do not alter kinetics of contraction.

Baseline kinetics of contraction of adult rat apical cardiomyocytes with upregulation of miR-16 and miR-26a showing time to peak (**A)**, time to 50% relaxation (**B**) and time to 90% relaxation (**C**). n/N=30/6 where ‘n’ is equal to the number of cardiomyocytes and ‘N’ represents number of rats. Data is displayed as mean ± SD and compared using one-way ANOVA.

|  **E**  **6A**  **B**  **C**  **D**  **F**  **G**  **H**  **I** |
| --- |

**Online Figure 6.** miR-16 and miR-26a do not change time to peak or time to 50% decay, and do not alter fractional release, or rates of SERCA and NCX.

Calcium fluorescent imaging of miR-16 and miR-26a transfected adult rat apical cardiomyocytes. **A** Time to peak calcium of transfected cardiomyocytes (control=66/4, miR-16=32/4 and miR-26a=23/4). **B** Time to 50% decay of calcium transient from peak (control=66/4, miR-16=31/4 and miR-26a=23/4). **C** Time to 80% decay of calcium transient from peak (control=66/4, miR-16=31/4 and miR-26a=23/4). **D** Rate of calcium transient decay fitted with a mono-exponential function (control=64/4, miR-16=30/4 and miR-26a=22/4). **E** Fraction release of calcium, where this is the ratio between calcium transient amplitude and caffeine-induced calcium transient amplitude (control=32/4, miR-16=22/4 and miR-26a=17/4). **F** SERCA rate (control=39/4, miR-16=20/4 and miR-26a=18/4). **G** NCX rate (control=24/4, miR-16=13/4 and miR-26a=9/4). **H** Rate of slow mechanisms of calcium transient decay (control=21/4, miR-16=8/4 and miR-26a=13/4). **I** Contribution of SERCA, NCX and slow mechanism rates to overall calcium transient decay. Data are displayed as mean ± SD for A-E and mean ± SEM for F-H, with significance determined by one-way ANOVA using Tukey’s post-hoc test and shown as follows: *=P<0.05, **=P<0.01.

|  **7A**  **B**  **C**  **D**  **E**  **F** |
| --- |

**Online Figure 7.** miR-16 and miR-26a do not alter cardiomyocyte β2AR response.

β_2_AR response (1µM isoprenaline in the presence of 300nM of the β_1_AR antagonist CGP201712A) in adult rat apical cardiomyocytes treated with miR-16 and miR-26a. **A** and **B** The peak contractile response following β_2_AR stimulation. **C** and **D** The fold increase in contractility after β_2_AR response. **E** and **F** The absolute increase in contractility (percentage shortening minus baseline) following β_2_AR stimulation. n/N = 6/6 for miR-16 experiments (**A**, **C** and **E**) and 10/10 for miR-26a (**B**, **D** and **F**). Numbers are displayed as n/N, where ‘n’ is equal to the number of cardiomyocytes and ‘N’ represents number of rats. Data are displayed as mean ± SEM and compared using Student’s t-test.

|  **8A**  **B**  **C**  **D** |
| --- |

**Online Figure 8.** Calcium current patch clamp of miR-16 and miR-26a transfected cardiomyocytes.

Patch clamp of miR-16 and miR-26a transfected adult rat cardiomyocytes. **A** LTCC current inactivation in apex (Control n/N=11/6, miR-16 n/N=9/6 and miR-26a n/N=7/4). **B** LTCC current inactivation in base (Control n/N=9/6, miR-16 n/N=9/5 and miR-26a n/N=8/5). **C** Recovery from inactivation in apex (Control n/N=8/6, miR-16 n/N=8/5 and miR-26a n/N=7/5). **D** Recovery from inactivation in base (Control n/N=6/5, miR-16 n/N=7/5 and miR-26a n/N=7/4). Statistical comparisons were made with RM-ANOVA. Data is displayed as mean ± SEM.


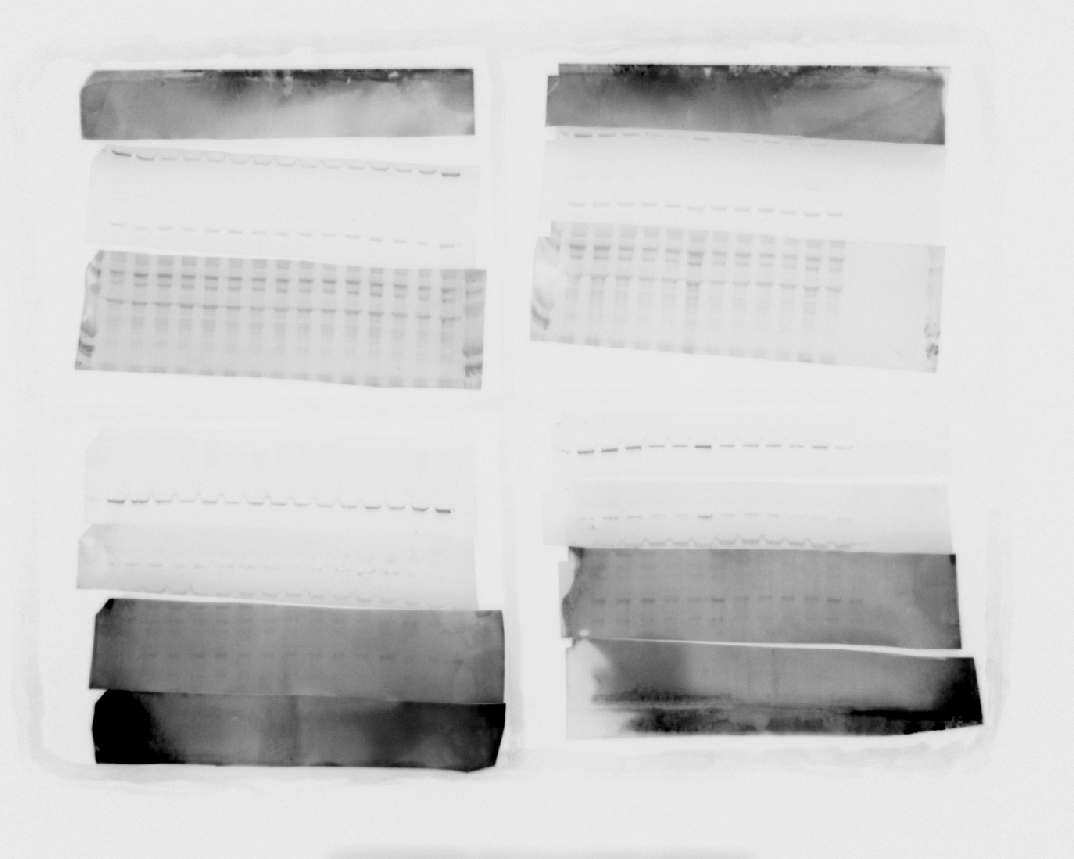

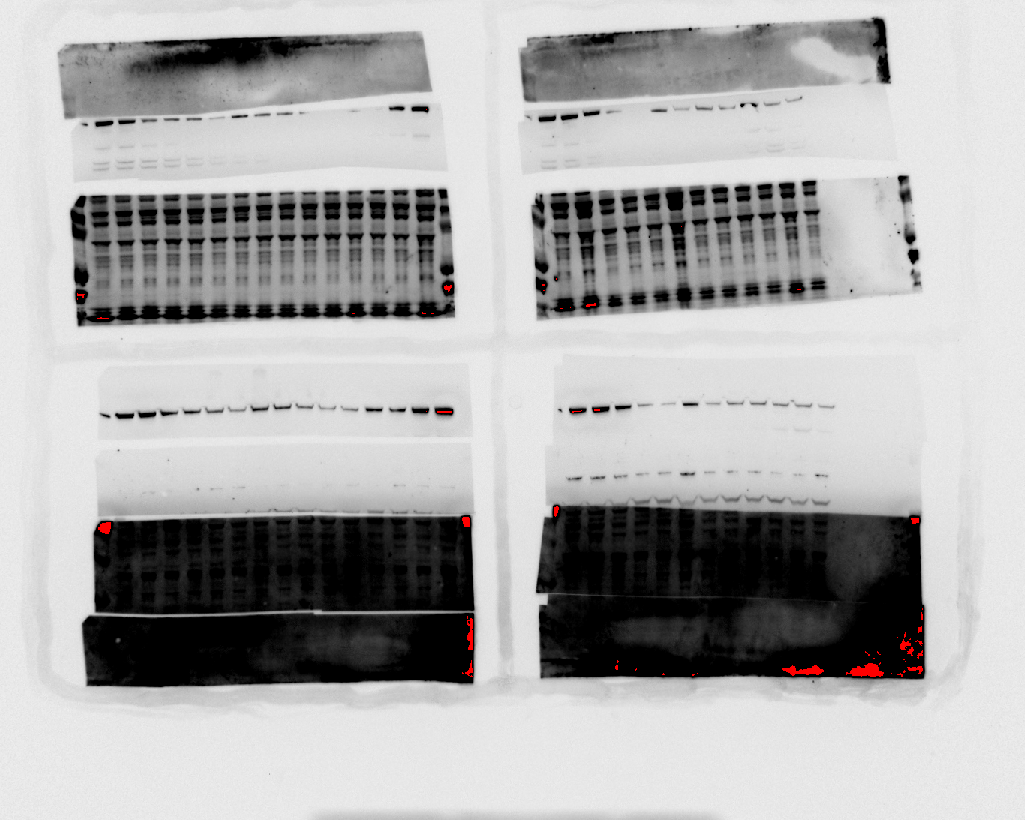

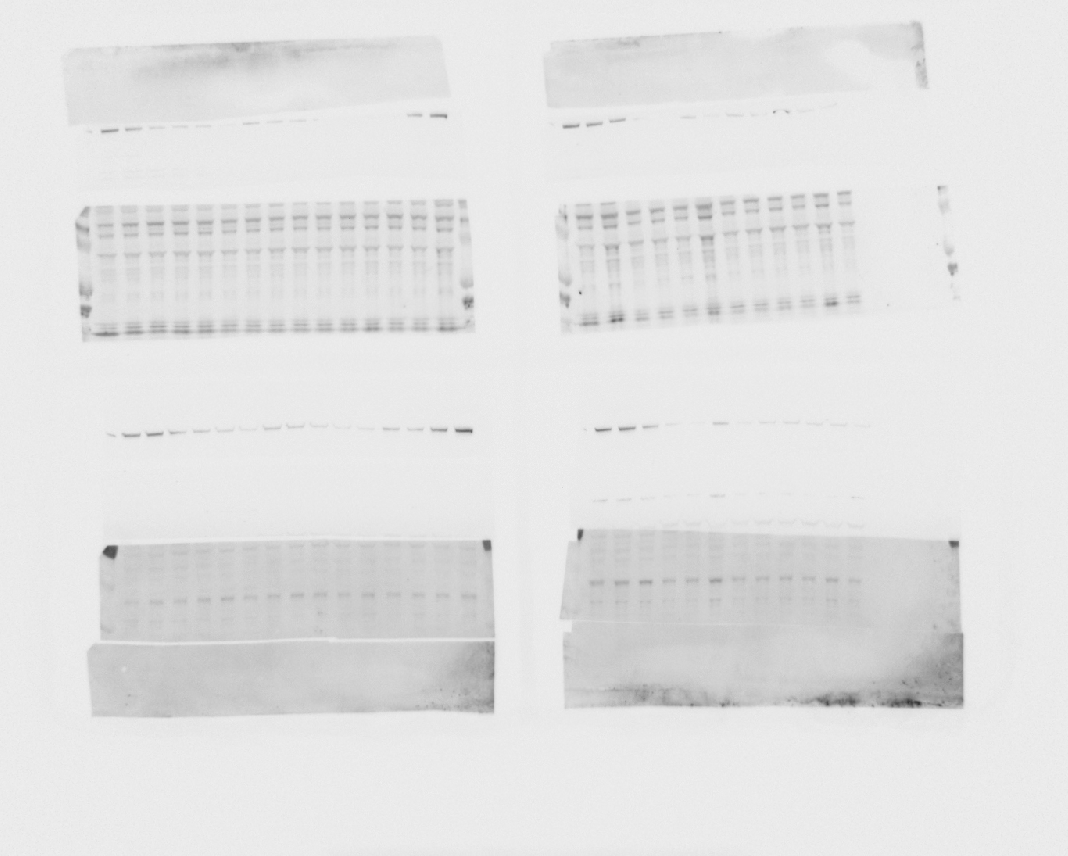

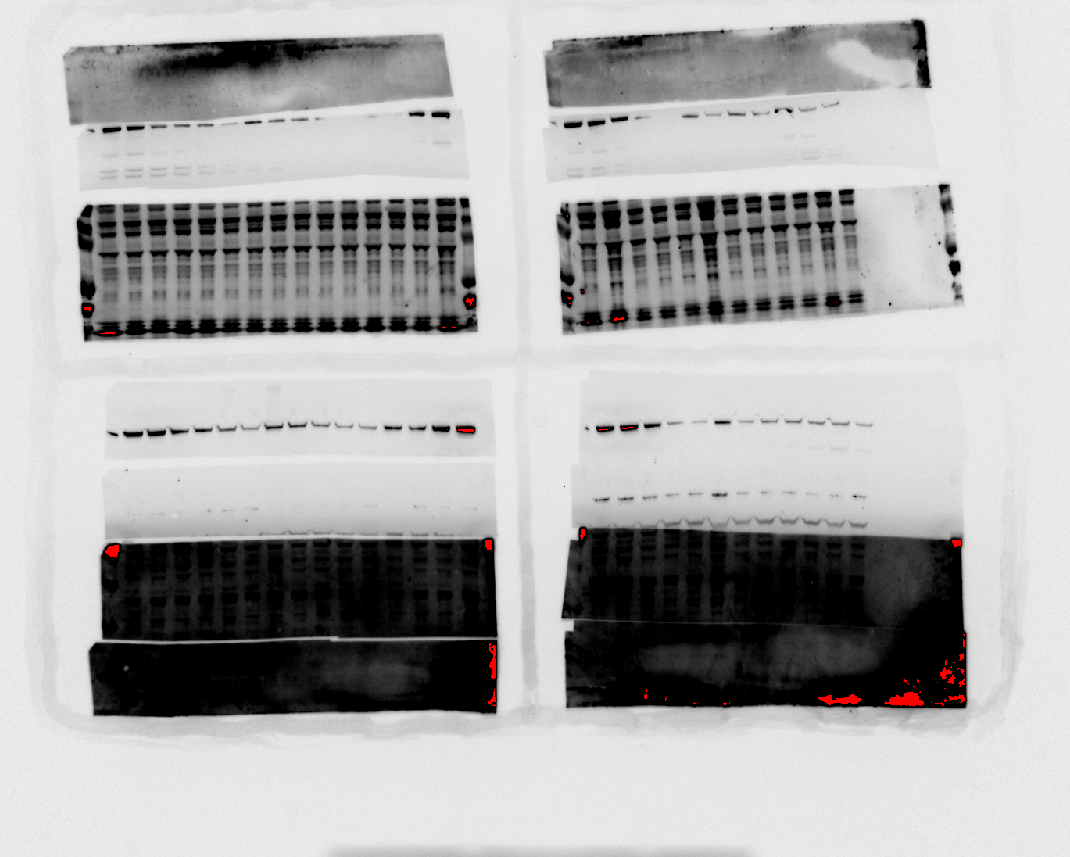


**B**

AAV-miR Vinculin

AAV-control Vinculin

AAV-miR CACNB1

AAV-control CACNB1

AAV-miR GNB1

AAV-control GNB1

AAV-miR RGS4

AAV-control RGS4


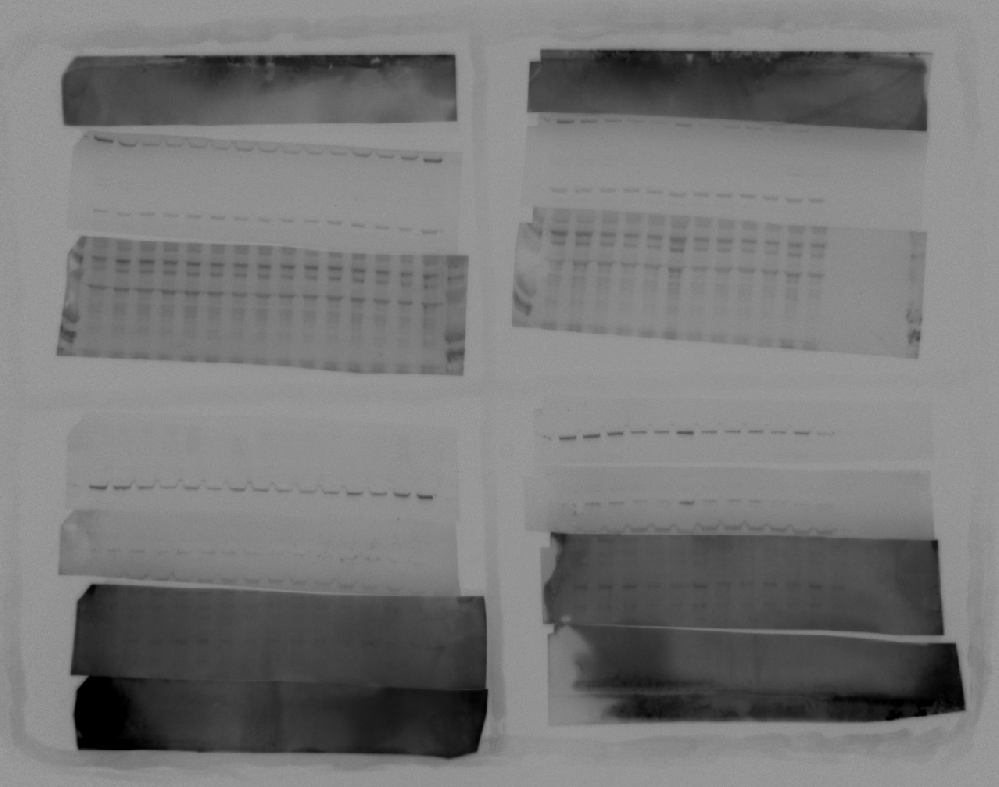


AAV-miR Vinculin

AAV-control Vinculin

AAV-miR CACNB1

AAV-control CACNB1

AAV-miR Vinculin

AAV-control Vinculin

AAV-miR GNB1

AAV-control GNB1

AAV-miR RGS4

AAV-control RGS4

**9A**

**Online Figure 9.** Original images of western blot gels.

**A** Single whole image of unedited gels shown at uniform exposure with regions of proteins shown. **B** Partitioned image of unedited gels shown at correct exposures for each protein. Bands used for the representative image in Figure 5J are underlined in blue.

|   **1A**  **B** |
| --- |

**Online Table 1.** Epinephrine concentration-response curves statistics

Epinephrine concentration-response curves for miR-16 and miR-26a treated cardiomyocytes (**A**) and in combination (**B**). Significance with non-linear regression comparing agonist *versus* response by F-test (bottom and top plateaus and logEC50).

**2A**

| **Hsa-miR-16** | **Protein** |
| --- | --- |
| ADCY1 | Adenylate cyclase type 1 |
| ADRA2A | Alpha-2A adrenergic receptor |
| ADRB2 | Beta-2 adrenergic receptor |
| ADRBK1 | G-protein coupled receptor kinase 2 (GRK2) |
| CACNA1B | Voltage-dependent N-type calcium channel subunit alpha-1B |
| CACNA1E | Voltage-dependent R-type calcium channel subunit alpha-1E |
| CACNB1 | Voltage-dependent L-type calcium channel subunit beta-1 |
| CACNB4 | Voltage-dependent L-type calcium channel subunit beta-4 |
| CDC42 | Cell division control protein 42 homolog |
| CREB5 | Cyclic AMP-responsive element-binding protein 5 |
| GNAI3 | Guanine nucleotide-binding protein G(k) subunit alpha |
| GNAL | Guanine nucleotide-binding protein G(olf) subunit alpha |
| GNB1 | Guanine nucleotide-binding protein G(I)/G(S)/G(T) subunit beta-1 |
| GNG12 | Guanine nucleotide-binding protein G(I)/G(S)/G(O) subunit gamma-12 |
| GRM4 | Metabotropic glutamate receptor 4 |
| GYS1 | Glycogen [starch] synthase, muscle |
| HTR4 | 5-hydroxytryptamine receptor 4 |
| ITPR1 | Inositol 1,4,5-trisphosphate receptor type 1 |
| MAP2K3 | Dual specificity mitogen-activated protein kinase kinase 3 |
| MAP3K4 | Mitogen-activated protein kinase kinase kinase 4 |
| MAPK3 | MAP kinase-activated protein kinase 3 |
| MKNK1 | MAP kinase-interacting serine/threonine-protein kinase 1 |
| PHKA1 | Phosphorylase b kinase regulatory subunit alpha, skeletal muscle isoform |
| PHKG2 | Phosphorylase b kinase gamma catalytic chain, liver/testis isoform |
| PRKAR2A | cAMP-dependent protein kinase type II-alpha regulatory subunit |
| PRKCA | Protein kinase C alpha type |
| PRKCZ | Protein kinase C zeta type |
| RGS3 | Regulator of G-protein signalling 3 |
| SNAP25 | Synaptosomal-associated protein 25 |
| SSR1 | Somatostatin receptor type 1 |
| SSTR3 | Somatostatin receptor type 3 |
| VAMP1 | Vesicle-associated membrane protein 1 |
| VAMP8 | Vesicle-associated membrane protein 8 |

**B**

| **Hsa-miR-26a** | **Protein** |
| --- | --- |
| ADCY2 | Adenylate cyclase type 2 |
| ADCY6 | Adenylate cyclase type 6 |
| ADRBK1 | G-protein coupled receptor kinase 2 (GRK2) |
| CACNA1C | Voltage-dependent L-type calcium channel subunit alpha-1C |
| CACNB2 | Voltage-dependent L-type calcium channel subunit beta-2 |
| CACNB4 | Voltage-dependent L-type calcium channel subunit beta-4 |
| CLTC | Clathrin heavy chain 1 |
| CREBBP | CREB-binding protein |
| GPSM1 | G-protein-signaling modulator 1 |
| GPSM2 | G-protein-signaling modulator 2 |
| GRM1 | Metabotropic glutamate receptor 1 |
| GSK3B | Glycogen synthase kinase-3 beta |
| ITPR1 | Inositol 1,4,5-trisphosphate receptor type 1 |
| MEF2C | Myocyte-specific enhancer factor 2C |
| MEF2D | Myocyte-specific enhancer factor 2D |
| MKNK2 | MAP kinase-interacting serine/threonine-protein kinase 2 |
| PLCB1 | 1-phosphatidylinositol 4,5-bisphosphate phosphodiesterase beta-1 |
| PRKCB | Protein kinase C beta type |
| PRKCD | Protein kinase C delta type |
| PRKCQ | Protein kinase C theta type |
| PRKX | cAMP-dependent protein kinase catalytic subunit PRKX |
| PYGL | Glycogen phosphorylase, liver form |
| RAP1A | Ras-related protein Rap-1A |
| RGS4 | Regulator of G-protein signaling 4 |
| SNAP29 | Synaptosomal-associated protein 29 |
| SSR1 | Somatostatin receptor type 1 |
| STX3 | Syntaxin-3 |
| TAB2 | TGF-beta-activated kinase 1 and MAP3K7-binding protein 2 |
| VAMP1 | Vesicle-associated membrane protein 1 |

**Online Table 2.** Predicted targets for miR-16 and miR-26a.

Targets of miR-16 (**A**) and miR-26a (**B**) proposed to influence cardiomyocyte contractility identified using miRWalk 2.0 and Panther DB.
